# Adolescent psychiatry of Y. Kasahara and succeeding research on *Hikikomori* in Europe and in Japan

**DOI:** 10.1002/pcn5.120

**Published:** 2023-07-18

**Authors:** Tadaaki Furuhashi

**Affiliations:** ^1^ Department of Psychopathology & Psychotherapy Graduate School of Medicine/Research Center of Health Nagoya Japan

**Keywords:** apathy syndrome, clinical classification of depression, *Hikikomori*, mood disorder, Yomishi Kasahara

## Abstract

This paper will focus on the works of one of Japan's representative psychiatrists, Yomishi Kasahara, particularly on his works in the 1970s in which he proposed the concept of student apathy, and will discuss how this work was carried over into a contemporary topic, the study of “*Hikikomori*.” Kasahara's well‐known paper “Clinical Classification of Depression” (Kasahara and Kimura, 1975) described the present state of patients with Type III as “they do not have a complete set of symptoms as in Type I, but sometimes show dependency, strong exaggeration, complication of other neurotic symptoms, little tendency of self‐reproaching, and tendency of accusing others”; the two subtypes as Type III‐1 “those that remain at the neurotic level” and Type III‐2 “those that transiently drop to the psychotic level.” We have summarized and introduced below the case presented in the paper with this Type III‐1. From today's perspective, where the concept of “*Hikikomori*” exists, this case could be considered as a typical case of “*Hikikomori*,” that is, a person with a tendency to avoid social roles and responsibilities and to immerse oneself in areas with no responsibilities, such as hobbies. “*Hikikomori*” was discovered in the late 1980s, but to be precise, it was just that the concept emerged. The same clinical condition had already been brilliantly found by Kasahara in the 1970s under the concept of “apathy syndrome,” which was distinguished from depression.

## INTRODUCTION

“*Hikikomori*” was discovered in the late 1980s. But what does that mean? This paper will focus on the works of one of Japan's representative psychiatrists, Yomishi Kasahara (born in 1928), particularly on his works in the 1970s in which he proposed the concept of student apathy, and will discuss how this work was carried over into a contemporary topic, the study of “*Hikikomori*.”

### Is apathy syndrome a mood disorder?

After graduating from Kyoto University School of Medicine in 1952, Kasahara continued his psychiatric clinical practice in Kyoto, and from 1968 he was engaged in the mental health care of Kyoto University students. There, he realized there were students who showed a specific apathy against studying and advocated a concept called student apathy. He also developed a technique called small psychotherapy to complement pharmacotherapy for depression. In 1972, Kasahara was appointed as a professor of the Department of Psychiatry, Nagoya University School of Medicine. He was extremely sensitive to the changes of the times, and in the 1990s, he wrote a book entitled *From Outpatient Psychiatry*, in which he described outpatients with mild depression who visited the university hospital.^1^ After his mandatory retirement, he continued to practice psychiatry in Nagoya, and continues to have a major influence on psychiatrists not only in Nagoya but all over Japan.

The word apathy (apathie) is derived from the Greek word apátheia (indifference, impassiveness), which is a word formed with the negative prefix “a” in front of the Greek word páthos (pathos, feelings). From just this, apathy syndrome appears to be a mental disorder in the mood disorders, however, its positioning has changed over time.

The concept of student apathy (1961), which had already been reported in the United States by Walters,^2^ was introduced to Japan in the 1960s. Thereafter, from the late 1960s to the 1970s, Kasahara pointed out the existence of similar symptoms among university students in Japan, which were also referred to as “retreat neurosis.”^2^ Kasahara indicated that this phenomenon was caused by the lengthening of the adolescent period due to social and economic changes.^3^ In fact, cases of businessmen had already been recognized at that time, and Kasahara stated that “it can no longer be called student apathy,”^3^ and called it “apathy syndrome.”^3^

Apathy syndrome has the following features.^3^ The following are the major three among the seven characteristics ([4–7] are not included here, as they are characteristics related to treatment and the course). (1) Subjectively, the patients are only aware of their apathy, lethargy, impassiveness, and loss of purpose of life, goal, and career path. They do not have ego‐alien experiences, such as anxiety, fret, depression, agony, and regret as in textbook neurotic patients. Therefore, they naturally lack the motivation to seek treatment of their own accord. (2) Objective behavior is aptly described as “retreat” or “escape” from the world. They rarely form painful subjective experiences “inside” themselves as “symptoms,” and just “act‐out” “externally.” However, the acting‐out is “negative acting‐out,” such as lethargy, retreat, and betrayal. Retreat is mainly retreat from their normal main business of life (in the case of students, studying), and often they do not show much resistance to taking part in areas of life other than their main business. This is related to hypersensitivity to superiority and inferiority or victory and defeat. They avoid anticipated defeat and humiliation. (3) Before suffering from the disease, they were rather overly well‐adjusted people. However, in a broad sense, they have an obsessive–compulsive personality. The common features are black‐or‐white dichotomous perfectionism, lack of aggression and lack of vigor. However, they do not show obsessive–compulsive symptoms in their medical history.^3^

Kasahara apprehended the pathology of this apathy syndrome with a certain broadness, from a neurotic level to a borderline case‐like level.^3^ Of course, Kasahara did not conclude from the beginning that the clinical unit he extracted as apathy syndrome should be apprehended with a broad pathology, but rather, in several research papers, he first tried to consider it as a form of depression. The flow is briefly shown below.

The first paper was co‐authored with Miyata and Yura titled “Neurotic Depressive States of the Recent Days,” in which they synthesized and compared the cases of a clinic in a certain area of Kyoto city, the cases of a neurology department of a corporate entity general hospital, and the cases Kasahara consulted, the outpatients of Kyoto University Department of Psychiatry.^4^ In the paper, Kasahara summarized several characteristics of the patients with depression who visited the outpatient clinic of Kyoto University Department of Psychiatry between January 1966 and May 1968 as follows. (1) Many are mild cases who have sufficient symptoms of depression but are mild in severity. The main characteristics of this mild type of depression are as follows: Many have typus melancholicus (Tellenbach, H.) or immodithymie (Shimoda, Hirasawa). Borrowing phrases from the dynamics group, many of them have an obsessive–compulsive personality defense in the interval, they have a common organizational characteristic in their disease onset situation, medication goes without saying but resting is more effective for treatment, and the required duration of treatment is short. (2) There are not many severe cases. (3) So‐called “masked depression,” that is, depression with a stronger psychosomatic tendency and without typical psychiatric symptoms, is somewhat different from the above‐stated mild depression, in which psychiatric symptoms are typical, although the severity is mild.^4^

Furthermore, according to Kasahara et al., the overall increase of cases with depression at that time, including cases in the neurology department of a corporate entity general hospital, could be considered as an increase of cases with neurotic conditions in patients with melancholy personalities. Kasahara presented one of the reasons for such a trend based on his clinical experience in mental health care for students at Kyoto University since 1968. It was because “typus melancholicus” itself had already drastically increased as one of the personality defenses against the difficulties of modern life. Furthermore, their paper had already predicted that “apathy” would replace “depression” as the leading neurological disorder in the future.^3^

In fact, Kasahara extracted the neurotic apathetic state of long‐term repeaters among the university students who had the above‐mentioned characteristics at that time as a single clinical unit in his second paper written in the same year as the first, 1971, entitled “‘Apathy’ specific to university students.”^5^

However, it can be said that it is due to Kasahara's own conscience as a person engaged in medical care that the conclusion for the question of whether this apathy state is a form of depression is still not drawn at this point in time. Actually, Kasahara positioned this apathy state 4 years later in his well‐known paper “Clinical Classification of Depression”^6^, ^7^ (a summary of the contents of this paper was translated into English by Kasahara himself in 1994) as Type III “Conflict‐reactive depression” among the six types of depression: Type I “Characterogenic (situational) reactive depression (Depressive episode, unipolar, mild severity),” Type II “Cyclothymic depression (Depressive episode, bipolar, recurrent),” Type III “Conflict‐reactive depression (So‐called neurotic, chronic),” Type IV “Pseudo‐cyclothymic schizophrenia (Atypical features [dysphoric, apathetic, impulse dyscontrol]),” Type V “Grief reaction (Repetitive thought of ‘loss’ or ‘event’),”and Type VI “Other depressive states (Unspecified [organic depressive syndrome, depressive states in childhood, adolescence, and in senile persons, and in others]).”^6^, ^7^

The paper described the present state of patients with Type III as “they do not have a complete set of symptoms as in Type I, but sometimes show dependency, strong exaggeration, complication of other neurotic symptoms, little tendency of self‐reproaching, and tendency of accusing others”; the two subtypes as Type III‐1 “those that remain at the neurotic level” and Type III‐2 “those that transiently drop to the psychotic level”; the premorbid personality as “immaturity, little love for orderliness and little concern for others”; and outbreaking situations as “excessive burden, difficulties relevant to personality defaults, interpersonal conflicts, maturational crisis”; attitudes toward treatment as “antidepressants are almost ineffective, intensive psychotherapy is needed”; clinical course as “they have a strong trend of becoming chronic and prolonged”; age as “two age ranges, one from late teens to 20s, the other in 40s or 50s”; physical features as “no characteristics”; life history as “already showed neurological symptoms or tendency of personality neurosis before onset of depression”; family image as “no characteristics”; provisional name as “conflict‐reactive depression”; and the relation with conventional diagnoses as “neurotic depression, depressive neurosis, reactive depression, psychogenic depression, psychogenic reaction, menopausal (regressive) depression, claiming depression, hysterodepression.”^6^, ^7^

Now, let me summarize and introduce below the case presented in the paper with this Type III‐1.^6^

(Patient) A male college student who was 20 years old at the time of initial examination

(Premorbid personality) He was serious, introverted, relatively enthusiastic, but not meticulous. He was not sociable and liked to be alone. He did not have many friends. He had been long asking himself the question, “What am I?”

(Medical History) After entering university, he spent 1 year as a class committee member and student activist. When the student movement settled down for the time being, he attempted to commit gas suicide and was admitted to a mental hospital in his hometown, where he spent 6 months under the diagnosis of depression (at the age of 20). In the following year and the year after that, he was hospitalized for depression, emptiness, pessimism, and suicide attempts for short periods (1 month and 2 months, respectively). When he was 22 years old, he visited his then doctor in charge. He was depressed and repressed in appearance, and subjectively complained a vague sense of emptiness and pessimism. However, there was nothing introspective or eccentric about him at all, and he described his internal agony with precise words…. After a preliminary interview, weekly interviews were continued for 3 years…. Along with the treatment, he became aware of his ambivalent feelings toward his parents, especially his father, which were held back deep inside him. Positive transference to the therapist occurred, and the patient became dependent on him. … The patient became reality‐oriented and began to work part‐time. … Religious interest arose. Through churchgoing, he had contact with a woman for the first time and also experienced a heartbreak. Three years later, he was almost completely freed from depression, emptiness, and pessimism.

(Course) The patient graduated university 3 years late. Not wanting to work in government offices or companies where people who graduated the same faculty as the patient wanted to work, he became a junior high school teacher and engaged in education for disabled children. At the age of 30, he became a quiet, diligent young teacher trusted deeply by parents.

From today's perspective, where the concept of “*Hikikomori* (social withdrawal)” exists, this case could be considered as a typical case of “*Hikikomori*,” that is, a person with a tendency to avoid social roles and responsibilities and to immerse oneself in areas with no responsibilities, such as hobbies. It can be said that Kasahara was a man of keen insight since he had felt that there was something in the patient which was beyond the category of (conflict‐reactive) depression although he had tried to fit him in it in an era when the concept of “*Hikikomori*” did not exist.

Furthermore, in his paper written in 1977, “Depression in Teenagers,” Kasahara and Ooi presented “new types of neuropsychiatric conditions” that needed to be distinguished from depression (apathy syndrome is one of them).^8^ In other words, it means that the aforementioned Type III “conflict‐responsive depression” and apathy syndrome are not necessarily the same. In this paper, Kasahara and Ooi wrote, “Those with Type III depression persistently and repeatedly demand provisions of love from the external world and others and show active interests as if they were trying to extort love, while those with apathy syndrome rather keep a certain distance and retreat from the offers of love from the outside world. They only slightly and timidly try to recover their relationship with the outside world only in places where they feel there are kinds of tenderness and warmth appropriate for themselves.”^8^ In this paper, the same case of Type III‐1 discussed in the paper of Kasahara et al. written in 1971^4^ is addressed as a case of apathy syndrome, distinguished from “conflict‐responsive depression.”^8^

As shown above, his success of extracting apathy syndrome as a clinical form, after clinically questioning whether apathy syndrome was a form of depression throughout the 1970s, was one of Kasahara's major works.

### Emergence of the term “*Hikikomori*”

Thereafter, in the late 1980s, the term “*Hikikomori*” appeared. There are several views on who coined the term “*Hikikomori*,” including a newspaper reporter or Fujiya Tomita, a clinical psychologist. Tomita has been engaged in counseling activities for truants since the 1980s, but was surprised at first by the number of consultations he received from parents with children over the age of 20. Tomita said, “It is only in these five years or so that I have come to be conscious of the term ‘*Hikikomori*.’ It has been more than a decade since I have been involved with parents and children suffering from refusal to attend school or find employment, but recently I encounter more and more young people, including these children, who have been rejecting relationships with others for a long time, as if they were ‘rejecting people’.”^9^ This expression gives out almost the same image we get from the present term, “*Hikikomori*.”

Saito defined social withdrawal as “a state of not participating in society for more than six months, not attending school or working, and having no intimate interpersonal relationships other than with family members.”^10^ Although some of the *Hikikomori* go out, it is considered that they stay at home in general. In fact, many social withdrawals spend much of their time in bed or on the couch and go out only late at night or early in the morning to avoid meeting their classmates or neighbors. Some go out every day, walk down the streets or take the train, pretending to go to school or work in an attempt to hide their condition. Saito, in fact, pointed out the similarities between “apathy syndrome” and some mild *Hikikomori* in this work.^10^ Saito distinguished between “*Hikikomori*” and “withdrawal” caused by depression or schizophrenia by showing these specific behavioral patterns. Saito stated that “*Hikikomori*” are found in the middle to upper class families and rarely seen in broken families, and that *Hikikomori* is not a disease or illness but a condition, and has its cause in the dysfunction of the communication system among the individual, family, and society.^10^

According to the guidelines of the Japanese Ministry of Health, Labour and Welfare, *Hikikomori* (social withdrawal) is “a phenomenon characterized by avoidance of social participation and isolation in one's home for 6 months or longer as a result of various elements. Although *Hikikomori* is a nonpsychotic phenomenon, actually, in some cases, undiagnosed schizophrenia may be involved.”^11^

The “*Hikikomori*” defined above is almost the same as today's “mild *Hikikomori*,” but as mentioned above, it can be said that apathy syndrome found by Kasahara also indicated a pathology included in this “mild *Hikikomori*” whose similarities with “apathy syndrome” were noted by Saito.^10^ Starting with the discovery of withdrawn adolescents in France in 2013, the existence of adolescents similar to “*Hikikomori*” has become evident around the world from around 2015. Let us take a look at these studies.

### Spread of “*Hikikomori*” across Europe

In Europe, interest in “*Hikikomori*” is growing significantly. It is not just an increased interest, but numerous academic papers have also been published, for example, in Spain and French Canada. When we searched for the word “*Hikikomori*” (only in the titles/chapters) on the French literature search site CAIRN, there were 23 papers (limited to papers published by February 2023). First, in the special feature issue on *Hikikomori* in the journal *Adolescence* in 2015, Vellut discussed the characteristic state of “une preference négative” in her first paper on “*Hikikomori*,” and the relationship between home withdrawal and the digital environment of PC screens in the other paper.^12^ Furuhashi introduced the Japanese withdrawal phenomenon in a journal with a similar special feature on *Hikikomori* in 2015, and posed a question whether it would be possible to treat *Hikikomori* patients, who do not come themselves for consultation to medical institutions, on the phone or online.^13^ In another 2017 paper, he presented another question as to why there was no one who committed suicide among the 300 or more *Hikikomori* patients the author treated.^14^ Tajan stated in a paper in 2014 that the representation of *Hikikomori* is associated with the helplessness of the parents, and is systematically linked to the Japanese school system, which would put particularly strong pressure on the pupils.^15^ He then discussed the characteristic state of *Hikikomori* in adolescence in a journal with a special feature on *Hikikomori* in 2015.^16^

Among the English‐language papers on the emergence of *Hikikomori* in each country, the countries, especially in Europe, included Spain, France, Ukraine, Italy, and Croatia. Italy was one of the first countries to introduce the Japanese “*Hikikomori*” phenomenon in a paper in Italian.^17^ Ranieri not only introduced the “*Hikikomori*” phenomenon, but also presented two concrete cases in Italy (both 13‐year‐old girls) in his paper in 2015, but there is a possibility that they were school refusals rather than “*Hikikomori*.”^18^ Many of the patients of the cases taken up as “*Hikikomori*” in Europe used to be patients in their teens, but recently, at last, patients in their thirties or forties have begun to be addressed. Even so, the paper by Ranieri et al. in 2018 took up a 15‐year‐old school refusal girl.^19^ A Croatian paper about the relationship between “*Hikikomori*” and Internet addiction was written in 2019.^20^ It first introduced general “*Hikikomori*” in Japan and said that *Hikikomori* is spreading like a silent epidemic all over the world.^20^ Then a case of a 24‐year‐old man was described in detail as a case of Southern Europe.^20^ According to the description, the case patient had no developmental disability or trauma in childhood. He lived with his parents and had not participated in society for at least 3 years.^20^ Although he had no other apparent mental illnesses or physical problems, he spent about 20 h a day surfing and playing games on the Internet.^20^ Furthermore, in 2017, Brazilian psychiatrists Gondim et al. wrote a paper on an interesting case of a Brazilian man who lived with his parents.^21^ He had withdrawn himself at home for 29 years from the age of 25 years old with no symptoms, such as hallucination or delusion, and had never seen a psychologist.^21^ He stored up philosophy books and was absorbed in reading them.^21^ The similarities to the Japanese cases are pointed out in the paper.^21^ Rooksby et al. pointed out that fear of infection, job loss, and social disruption due to lockdown rules because of COVID‐19 could also add to the risk of persistent social withdrawal.^22^

Regarding the relationship with Internet addiction, there are papers such as a survey paper on the association between “commuting (business and private), life satisfaction, stress and (over‐) use of the Internet” in 5,039 adolescents in the United Kingdom^23^; a paper reviewing literature on neurobiological, psychosocial, and clinical positions of Internet addiction taking into account factors such as the diagnostic criteria, nosological labels, and rating scales^24^; and a paper that evaluates items including depression, anxiety, interpersonal susceptibility, and hostility in 500 Taiwanese students using the Symptom Checklist‐90 item‐Revised Scale.^25^ There is also a paper that mainly evaluated “the relationship between traumatic experiences and Internet addiction symptoms, with negative automatic thoughts or low self‐esteem as mediators of these relation” in 112 adolescents in South Korea^26^; a paper that investigated 3,289 junior high school students about “self‐esteem and loneliness mediating the link between family functioning and adolescent Internet addiction”^27^; a paper that established the diagnostic classification test for Internet addiction (the DCT‐IA) using the diagnostic classification model based on the DSM‐5, and the latest psychometric method^28^; and a paper that proves that Internet addiction tends to have a complication of depression.^29^

## CONCLUSIONS

“*Hikikomori*” was discovered in the late 1980s, but to be precise, it was just then that the concept emerged. The same clinical condition had already been brilliantly found by Kasahara in the 1970s under the concept of “apathy syndrome,” which was distinguished from depression in relation to depression. This is closely related to the question of whether *Hikikomori* should be considered as a disease now, and if so, in which category it should be placed.

## CONFLICT OF INTEREST STATEMENT

The author declares no conflicts of interest.

## ETHIC APPROVAL STATEMENT

This paper does not use personal infomation involving an ethics statement.

## PATIENT CONSENT STATEMENT

N/A.

## CLINICAL TRIAL REGISTRATION

N/A

## Data Availability

This paper does not use data.
